# Childhood Maltreatment Alters the Neural Processing of Chemosensory Stress Signals

**DOI:** 10.3389/fpsyt.2020.00783

**Published:** 2020-08-06

**Authors:** Ayline Maier, Luca Heinen-Ludwig, Onur Güntürkün, René Hurlemann, Dirk Scheele

**Affiliations:** ^1^Division of Medical Psychology, Department of Psychiatry and Psychotherapy, University Hospital Bonn, Bonn, Germany; ^2^Department of Psychology, Laboratory for Biological Psychology, Ruhr-University of Bochum, Bochum, Germany; ^3^Department of Psychiatry, School of Medicine & Health Sciences, University of Oldenburg, Oldenburg, Germany; ^4^Research Center Neurosensory Science, University of Oldenburg, Oldenburg, Germany

**Keywords:** amygdala, childhood maltreatment, fMRI, hypervigilance, olfaction, oxytocin

## Abstract

Accumulating evidence suggests that childhood maltreatment (CM) confers risk for psychopathology later in life by inducing hypervigilance to social threat cues such as fearful faces. However, it remains unclear whether the modulatory impact of CM extents to the olfactory domain of social communication in humans. To address this question, we examined whether CM modulates the neural processing of chemosensory threat signals in sweat and whether CM affects the stress-reducing effects of oxytocin (OXT) in this context. In a randomized, double-blind within-subject functional MRI study design, 58 healthy participants (30 females) received intranasal OXT (40 IU) or placebo (PLC) and completed a forced-choice emotion recognition task with faces of varying emotion intensities (neutral to fearful) while exposed to sweat stimuli and a non-social control odor. Axillary sweat samples were collected from 30 healthy male donors undergoing an acute psychosocial stressor (stress) and ergometer training (sport) as control in a pre-study. CM was assessed by the 25-item Childhood Trauma Questionnaire (CTQ). The final fMRI analysis included 50 healthy participants (26 females). Regression analysis showed a stress-specific association of CTQ scores with amygdala hyperreactivity, hippocampal deactivation, and increased functional connectivity between the amygdala and the hippocampus, medial orbitofrontal cortex, and the anterior cingulate cortex (ACC) under PLC. Furthermore, we observed a positive association of CTQ scores and the dampening effects of OXT on stress-related amygdala responses. Our findings suggest that CM may induce hypervigilance to chemosensory threat cues in a healthy sample due to inefficient frontolimbic inhibition of amygdala activation. Future studies should investigate whether increased recruitment of the intralimbic amygdala-hippocampus complex reflects a compensatory mechanism that prevents the development of psychopathology in those who have experienced CM. Furthermore, the results reveal that the stress-specific effects of OXT in the olfactory domain are more pronounced in participants with increasing levels of CM exposure.

## Introduction

Childhood maltreatment (CM) presents a leading risk factor for the later development of psychopathology (1), with CM exposure accounting for over 30% of adult-onset psychiatric disorders (2). Recent efforts to identify etiological mechanisms that mediate this association, suggest CM experiences become biologically embedded (3) in altered trajectories of neurodevelopment (4) and behavior (5). Specifically, burgeoning data underscore the notion that a history of CM is linked to changes in sensory systems (6) and the neural circuitry underlying emotion regulation and threat responsivity (4).

One of the most frequently reported neuroimaging finding in individuals with a history of CM is exaggerated amygdala reactivity to threatening faces (fearful and angry) (7–9). Furthermore, individuals with a history of CM exhibit increased amygdala functional connectivity (FC) with the anterior cingulate cortex (ACC) (10) and with regions of the prefrontal cortex (PFC), in particular the orbitofrontal cortex (OFC) (11) during the exposure to threatening faces. The amygdala represents a key node in threat detection and in the coordination of adaptive behavioral and autonomic responses to these threat signals (12). Aberrant amygdala activations are observed across psychiatric disorders (13) and the amygdala threat detection process has been suggested to mediate the relationship between CM and psychopathology later in life (14, 15). Both the ACC and the OFC feature reciprocal functional and anatomical connections with the amygdala (16) and co-activations of the ACC and OFC with the amygdala are central to efficient emotion regulation by enabling a down-regulation of amygdala reactivity to threatening stimuli (17–19). These findings show that CM is associated with a dysregulated threat circuitry manifested in a phenotypic hypersensitivity towards social threat cues. However, it remains unclear whether the modulatory impact of CM extents to the olfactory domain of social communication.

Phylogenetically one of the most ancient senses, olfaction is essential for survival due to its alarm function. In humans, the ability to identify olfactory threat cues in the environment and respond to them in an adaptive manner is well developed (20). Olfaction plays a key role in the modulation of behavior and interpersonal relationships (21), with accumulating evidence indicating social chemosignaling in humans (20, 22–24). Human social chemosignals have been shown to convey information with respect to kin recognition (20), mother-infant bonding (25), disease detection (26), aggression (24) and emotional states (23). A recent line of research demonstrates that chemosensory communication of threat cues in axillary sweat modulates cross-modal emotion perception of ambiguous threatening facial stimuli and produces widespread neural threat responses in the amygdala, ACC, hippocampus, the prefrontal cortex, and fusiform face area (FFA) (27–30). These effects are even more pronounced in individuals with heightened stress vulnerabilities such as patients with anxiety disorders (31, 32). The olfactory system and the emotion circuitry are largely intertwined and share neuroanatomical pathways *via* the amygdala, hippocampus, and OFC (33). Thus, olfactory stimulation directly evokes emotions and autonomic responses *via* these pathways (34). Furthermore, there is evidence suggesting a separate representation of pleasant and unpleasant odors in the medial and lateral parts of OFC (35). The overlap of brain regions showing aberrant threat-induced activation patterns in CM studies and olfactory projection areas render the olfactory domain a potential pathogenic pathway following CM exposure. Recent findings have linked CM to altered activation in a widespread network of neocortial areas including the OFC and hippocampus during non-threatening olfactory stimuli presentation in females (36). Another study observed significant reductions of olfactory bulb volume and olfactory function in women with a history of CM (37). Moreover, olfactory dysfunctions and altered processing of non-social olfactory threat cues have been observed in individuals with post-traumatic stress disorder (PTSD) (38–40). However, whether CM modulates the processing of social olfactory cues remains unclear.

The hypothalamic peptide hormone oxytocin (OXT) has been increasingly recognized as a promising therapeutic candidate for stress-related disorders such as major depressive disorder and PTSD due to its role in stress regulation and social behavior (41). Animal models demonstrate long-term consequences of early life experiences in the oxytocinergic system, with rodents exhibiting lower OXT receptor expression in the amygdala and hypothalamus after receiving less maternal care (42, 43) and increased serum and hypothalamic OXT levels in maternal separation models (44). Likewise, human studies observed lower OXT concentrations in the cerebrospinal fluid of men (45) and women with a history of CM (46). Interestingly, a particularly strong effect was identified for emotional abuse. However, less severe forms of CM were positively associated with urine OXT levels in adults (47). In line with this, women with a history of sexual abuse during childhood exhibited higher blood OXT levels in response to a laboratory psychosocial stressor, i.e. the Trier Social Stress Test (TSST) (48) compared to controls (49). Furthermore, human intranasal administration of OXT enhanced the stress-buffering effects of social support during the TSST (50, 51) and we recently found that the peptide reduces amygdala reactivity to social chemosensory threat signals (27). Importantly, a plethora of studies observed that the effects of intranasal oxytocin vary as a function of social context and interindividual variables such as childhood experiences (52, 53). For instance, the stress-buffering effects of OXT after the TSST were only evident in women with higher levels of adverse childhood experiences (51), while the peptide had no significant effect on handgrip force in reaction to an infant crying in women with harsh parenting experiences (54). However, it remains to be investigated whether CM affects the effects of OXT in the context of social chemosensory threat cues.

Given the adverse behavioral and health consequences of CM (55, 56), there is a pressing need to identify neurobiological compensatory mechanisms that help individuals to maintain or rapidly regain mental well-being in the aftermath of CM (57). Notably, a significant proportion of individuals with a history of CM function well and are clinically resilient despite CM-induced neurobiological changes (4, 58). This suggests that additional neurobiological mechanisms may be present that enable these individuals to effectively compensate for CM-induced brain changes (4). Potential compensatory mechanisms for CM-associated hypervigilance have recently been examined in response to threatening facial stimuli revealing a heightened intra-limbic FC between the amygdala and the hippocampus in resilient adults (59). However, it remains unclear whether CM also modulates threat responsivity in the olfactory domain and which potential compensatory mechanisms may be observed in a resilient sample.

The current study consists of a secondary analysis utilizing an existing data set of a randomized, double-blind, placebo (PLC)-controlled trial by Maier et al. (27) that was collected to explore the oxytocinergic modulation of chemosensory communication of stress. The functional magnetic resonance (fMRI) study involved 58 healthy volunteers completing a forced-choice emotion recognition task with facial stimuli of varying emotion intensities (neutral to fearful) while exposed to sweat stimuli and a non-social control (raspberry odor) after intranasal PLC and OXT administration, respectively. Axillary sweat samples were obtained from healthy male donors undergoing an acute psychosocial stressor (stress) and ergometer training (sport) as control in a pre-study. In this secondary analysis, we investigated the modulatory effect of CM on the processing of chemosensory threat signals and whether CM affects the anti-stress effects of OXT in this context. The measure relevant to the current hypothesis was the Childhood Trauma Questionnaire (CTQ) (60). Our primary hypothesis was that CM would be associated with increased neural reactivity and increased frontolimbic as well as intralimbic FC to chemosensory threat signals. Secondary, we assumed that CM also modulates the effects of OXT on the processing of chemosensory stress cues.

## Methods

The original randomized, double-blind, PLC-controlled, within-subject, cross-over trial (*n* = 58) by Maier et al. (27) was conducted between 2015 and 2017 at the Division of Medical Psychology of the University of Bonn, Germany. The study methods were previously described in full detail (27) and are summarized here.

### Participants

The study sample included 58 healthy (26 females, mean ± SD age, 24.90 ± 3.11 years), right-handed, heterosexual, non-smoking volunteers recruited from the local population *via* online advertisement and public posting. The Mini-International Neuropsychiatric Interview (MINI) (61) was used to screen for a history of psychiatric or physical disease prior to study enrollment. Furthermore, participants were screened for anosmia using the Sniffin’Sticks test battery, which comprises an odor identification and discrimination test (Burghart GmbH, Burghart Wedel, Germany). Participants were lifetime naïve to prescribed psychoactive medication and none of the participants were pregnant or used hormonal contraceptives during the study. MRI contraindications were additional exclusion criteria. CM experiences were assessed using the 25-item retrospective CTQ (60). The CTQ measures five types of adverse childhood experiences: emotional neglect, emotional abuse, physical neglect, physical abuse, and sexual abuse (62). A 5‐point Likert scale is used for responses ranging from 1 (*never true*) to 5 (*very often true*) and scores ranging from 5 to 25. In addition, depressive symptoms within the previous 2 weeks and subjective anxiety for the past month were assessed using the Beck Depression Inventory–II (63) and the State-Trait Anxiety Inventory (STAI) (64). Autistic-like traits were measured *via* the Autism Spectrum Quotient questionnaire (AQ) (65). Demographic and psychometric sample characteristics are listed in **Table 1**. Eight participants had to be excluded from the fMRI analysis due to technical malfunctions or excessive head motion (>3 mm/°) during scanning, leaving 50 participants (26 females, mean ± SD age, 24.54 ± 3.09 years) for the fMRI data analyses.

**Table 1:** Demographic and psychometric sample characteristics.

	Mean ± SE (Range) (*N* = 58)
Age (years)	24.9 ± 0.41 (19–31)
Sex (F/M)	30/28
Education (years)	16.83 ± 0.38 (12–25)
CTQ sum score	33.98 ± 0.75 (29–53)
CTQ emotional neglect	7.55 ± 0.35 (5–16)
CTQ emotional abuse	6.66 ± 0.29 (5–16)
CTQ physical abuse	8.66 ± 0.16 (6–13)
CTQ physical neglect	5.86 ± 0.18 (5–9)
CTQ sexual abuse	5.26 ± 0.22 (5–18)
BDI	2.09 ± 1.07 (0–12)
STAI Trait	31.45 ± 0.92 (22–52)
AQ	13.81 ± 0.65 (2–28)

Childhood maltreatment experiences were assessed by means of the CTQ.

CTQ, Childhood Trauma Questionnaire; BDI, Beck Depression Inventory; STAI, State Trait Anxiety Inventory; AQ, Autism Spectrum Quotient.

The study was conducted in accordance with the latest version of the Declaration of Helsinki and approved by the local ethics committee of the Medical Faculty of the University of Bonn. All participants provided written informed consent before screening and were reimbursed for participation.

### Study Design

In a randomized, double-blind, PLC-controlled, within-subject crossover design, participants received either OXT (Novartis, Basel, Switzerland) or PLC intranasally in two separate experimental sessions of at least 24 h apart. At the beginning of each fMRI testing session, participants self-administered a single intranasal dose of 40 IU synthetic OXT or PLC under the supervision of an experimenter following a standardized protocol (66) (5 puffs balanced across nostrils, at an inter-puff interval of 50 seconds to allow the solution to be absorbed into the nasal epithelium). The amount of administered substance was weighed and was supplemented by an additional puff if it fell below a set minimum (40 IU = 1000mg). The PLC solution contained identical ingredients except for the peptide itself. Functional MRI scanning started 30 min after nasal administration and was followed by an anatomical scan. Participants abstained from caffeine and alcohol intake for 24 hours prior to arrival. Participants’ olfactory functioning was verified after nasal spray administration in both scanning sessions using a staircase olfactory threshold test (Burghart GmbH, Wedel, Germany) [67) (for full description, see (27)].

### Olfactory Stimuli and Presentation

During the fMRI experiment, participants were exposed to three different olfactory stimuli: male axillary sweat obtained from an independent sample of 30 healthy donors (mean ± SD age, 23.30 ± 2.67 years) who underwent both the (i) TSST (stress sweat) (48) and (ii) ergometer training (sport sweat), and as a non-social control (iii) chemically synthesized raspberry (Burghart GmbH, Wedel, Germany) [for detailed description of stimuli generation, see **SI** and (27)]. Sweat donors experienced significantly greater stress during the TSST compared to the physical exercise condition manifested in elevated salivary cortisol levels and state anxiety ratings (27). Chemosensory stimuli did not exhibit detectable differences in odor quality between treatment scan sessions, which was validated by an independent sample of participants who rated the pleasantness, intensity, and familiarity of the stimuli (27).

Olfactory stimuli were administered *via* a three-channel, computer-controlled, MRI compatible air-dilution olfactometer (OG001, Burghart GmbH, Wedel, Germany). Odorant flows (5 lpm) were directed *via* 10* m* tubes through an odorless oxygen mask, which participants wore inside the scanner. At stimuli offset, participants breathed ambient air through the exhalation ports of the oxygen masks. The odor channels were triggered using a specialized proprietary olfactometer control software (OG Control, Burghart GmbH, Wedel, Germany).

### Respiratory Signal Recording

Respiratory compliance was monitored online throughout fMRI scanning *via* an MR-compatible chest-strap-based respiration transducer (Biopac, RX-TSD221-MRI) to ensure that inhalations (i.e. thoracic expansions) were temporally aligned with odor delivery. Respiration signals were recorded using a Biopac MP150 system and the accompanying AcqKnowledge Acquisition & Analysis Software (Version 4.3.1) applying a sampling frequency of 1000 Hz. Noise was removed by means of a hardware-based filter included in the amplifier with a low pass filter of 1 Hz and a high pass filter of 0.05 Hz.

### fMRI Task

For the fMRI scan, an adapted version of an established emotion recognition paradigm was utilized (29). In a forced-choice paradigm, male facial stimuli were briefly presented at four emotion intensity levels (neutral, low fearful, medium fearful, and high fearful). Participants were instructed to identify whether the stimuli depicted a neutral or fearful expression while they were exposed to stress sweat, sport sweat or raspberry (non-social control odor). Odor delivery *via* the olfactometer was synchronized with respiratory cues (green fixation cross) and participants were instructed to breathe orthonasally and inhale on cue throughout the experiment. In each trial, odor delivery spanned the duration of the inhalation cue (1300 ms) as well as the emotional facial stimuli (200 ms) for a total duration of 1500 ms and was preceded by an exhalation cue (red fixation cross, 2000 ms). Experimental trials were separated by a jittered inter-stimulus interval (black fixation cross, 4,000–6,000 ms) and a new trial started immediately after the response was recorded or after 2000 ms if no response was made. Each of the three olfactory stimuli were presented 48 times in a random order, resulting in 144 trials and an experiment duration of about 20 min (for full description of the fMRi task, see **SI** and (27).

### Image Acquisition

A Siemens MAGNETOM Trio MRI system (Siemens, Erlangen, Germany) operating at 3T and equipped with a 32-channel phased-array head coil (Siemens, Erlangen, Germany) was used to acquire T2*-weighted echoplanar (EPI) images with blood-oxygen-level-dependent contrast (TR = 2500 ms, TE = 30 ms, pixel size: 2 x 2 x 3 mm, slice thickness = 3.0 mm, distance factor = 10%, FoV = 192 mm, flip angle = 90°, 37 axial slices). High-resolution anatomical reference images were obtained on the same scanner using a T1-weighted 3D MPRAGE sequence (imaging parameters: TR = 1660 ms, TE = 2.54 ms, matrix size: 256 x 256, pixel size: 0.8 x 0.8 x 0.8 mm, slice thickness = 0.8 mm, FoV = 256 mm, flip angle = 9°, 208 sagittal slices).

### fMRI Data Analysis

Functional imaging data were realigned and spatially normalized to the standard Montreal Neurological Institute (MNI) space and smoothed (Gaussian kernel, 6mm FWHM) using SPM12 software (Wellcome Trust Centre for Neuroimaging, London, United Kingdom; http://www.fil.ion.ucl.ac.uk/spm) implemented in MATLAB R2010b (MathWorks, Natick, Massachusetts) [for further detail, see **SI** and (27)].

Onsets and durations of the 24 experimental conditions (treatment (PLC, OXT) × odor (stress, sport, raspberry) × emotion intensities (neutral, low fearful, medium fearful, high fearful) were modeled by a stick function convolved with a hemodynamic response function, with the trial onset defined as the onset of odor delivery. Respiratory noise correction was performed using the PhysIO toolbox (68). The movement parameters (realignment parameters) and respiratory noise regressors were included as nuisance regressors in the design matrix. For the fMRI statistical analysis, we used a two-level random-effects approach based on the general linear model as implemented in SPM12 [for full description, see **SI** and (27)].

On the group-level we performed multiple regression analysis. Due to the absence of specific neural effects of emotion intensity, chemosensory-induced responses were averaged across all intensity levels. The modulatory effect of CM on the processing of chemosensory threat signals was measured by regressing CTQ sum scores on the differential contrast between blood-oxygen-level-dependent (BOLD) signal response to stress relative to sport odor [(Stress _(PLC)_ > Sport _(PLC)_)]. To explore whether CM moderates the stress-specific effects of OXT on the processing of chemosensory threat signals, CTQ scores were regressed on neural responsiveness to the contrast [(Stress_(PLC)_ > Sport_(PLC)_) – (Stress_(OXT)_ > Sport_(OXT)_)]. Furthermore, we also tested potential modulatory effects of CM on the neural processing of the non-social odor (raspberry) by regressing CTQ sum scores on the BOLD signal response to the contrasts [(Raspberry_(PLC)_)] and [(Raspberry_(PLC)_ > (Raspberry_(OXT)_)]. The fMRI analysis focused on a set of *a priori* defined bilateral regions of interest (ROIs) consisting of the amygdala, hippocampus, ACC, FFA, lateral OFC (lOFC) and medial OFC (mOFC). All ROIs were anatomically defined according to the Wake Forest University PickAtlas, version 3.0. *P*-values were corrected for multiple comparisons (family-wise error (FWE)) based on the size of the ROI, and *P* < 0.05 was considered significant. Parameter estimates were extracted from significant clusters of the BOLD response analysis (for full description, see **SI**).

### Connectivity Analysis

To explore the modulatory effects of CM on the functional interplay of brain regions showing significant CM-associated changes in neural responsiveness to chemosensory stress cues in the BOLD analysis, we regressed CTQ sum scores on the FC between these regions and the *a priori* defined ROIs (amygdala, hippocampus, ACC, FFA, lOFC and mOFC). For this purpose, we carried out a generalized psychophysiological interaction [gPPI; (69)] in SPM12. Seed regions were identified as significant clusters of the BOLD analysis. All target ROIs were anatomically defined using the Wake Forest University PickAtlas, version 3.0. On the first level, hemodynamic deconvolution was performed on the extracted time series to remove the effects of the canonical hemodynamic response (HRF). The resulting time series were multiplied by the psychological variables and reconvolved with the HRF to obtain the PPI interaction terms. The gPPI analysis for each subject was performed on the first level and included the same task regressors as specified for the BOLD analysis. On the second level, we regressed CTQ sum scores on the FC between seed and target regions for the contrasts [Stress_(PLC)_ > Sport_(PLC)_] and [(Stress_(PLC)_ > Sport_(PLC)_) − (Stress_(OXT)_ > Sport_(OXT)_)]. Results were considered significant at *P*_FWE_ < 0.05 (peak-level inference) adjusted to the size of the ROIs. Given that FC is susceptible to small frame-to-frame head movements, we calculated the mean frame-wise displacement (FD) (70) for each subject in each session. The FD has been shown to have a strong association with motion-induced artifacts in functional connectivity (71). Results revealed that all subjects exhibited FDs below the recommended threshold for task-based FC of 0.9 mm (72) during scanning in both testing sessions, respectively.

### Statistical Analysis

Statistical analyses were conducted with SPSS, version 24 (IBM, Armonk, N.Y.). Linear regression analyses were performed to estimate the effect of CM on emotion recognition during axillary sweat presentation. For these regression models, CTQ sum scores were used as the predictor variable and the differences in fearful recognition ratings of the emotional facial stimuli (range: 0 faces rated as fearful - 12 faces rated as fearful) between the stress and the sport condition for each emotion intensity level (neutral, low fearful, medium fearful, and high fearful) served as the criterion variables, respectively. The resulting four regression analyses were performed for the PLC condition and the modulating effects of OXT (OXT < PLC). For the regression models testing the effect of CM on emotion recognition during the presentation of the non-social control odor raspberry, CTQ sum scores served as the predictor variable and fearful recognition ratings for each emotion intensity level functioned as the criterion variables, respectively. These four regression analysis were computed for the PLC condition and the modulating effects of OXT (OXT > PLC). Furthermore, we tested multiple regression models predicting CM-related behavioral (emotion recognition rating) and neural responses (extracted parameter estimates) by the five CTQ subscales in order to explore maltreatment-specific predictions in the current sample. Pearson’s product-moment was used for correlation analyses. Reported *P*-values are one-tailed for directional analyses and two-tailed for all non-directional analyses.

### Mediation and Moderation Analysis

To control for the influence of possible confounding variables on our observed CTQ-associated response pattern, moderation, and mediation effects were assessed for the covariates subjective anxiety, depressive symptoms, autistic-like traits, age, sex and education time using the PROCESS macro for SPSS, version 3.1 (model 1 and model 4) (73). For all regression analyses, CTQ sum scores served as the predictor variable, respectively. CTQ-associated differences in fearful recognition ratings of the emotional facial stimuli between the stress and the sport condition and parameter estimates extracted from significant clusters of the BOLD analysis to the contrasts [(Stress_(PLC)_ > Sport_(PLC)_)] and [(Stress_(PLC)_ > Sport_(PLC)_) − (Stress_(OXT)_ > Sport_(OXT)_)] served as the criterion variables, respectively. Using heteroscedasticity-consistent standard errors and mean-centering, the significance of indirect effects was examined using 95% bootstrapped (10,000 bootstrap samples) symmetric confidence intervals (95% CIs). Indirect effects were considered significant when the upper and lower bound of 95% CI did not contain zero. As the underlying mediation framework of PROCESS does not support dichotomous mediators, we explored a potential mediation effect of sex by employing the Baron and Kenny four steps regression approach (74). A moderation effect was assumed when the interaction term between the predictor variable CTQ and a moderation variable was significant. For these analyses the level of statistical significance was set at *P* < 0.05 and all reported *P*-values are two-tailed.

## Results

### Behavioral Results

**Figure 1:**
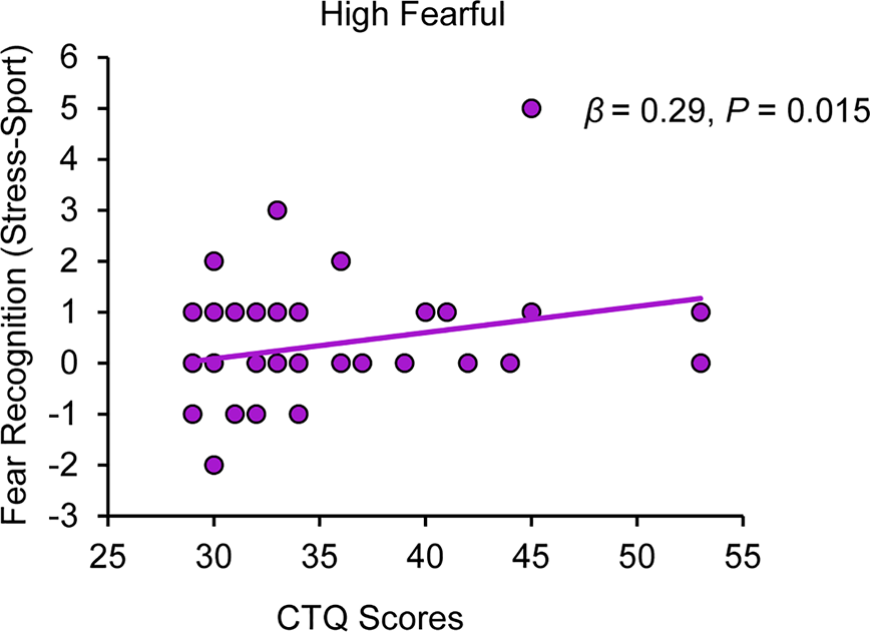
Childhood maltreatment and the impact of chemosensory stress signals on fear recognition. Childhood Trauma Questionnaire (CTQ) scores are positively associated with a stress odor induced bias in the recognition of high fearful faces (range: 0 faces rated as fearful - 12 faces rated as fearful). CTQ, Childhood Trauma Questionnaire.

Regression analyses revealed that CTQ sum scores were associated with an increased stress-specific recognition of high fearful faces under PLC, (*β* = 0.29, *P* = 0.015), with 8% of the variation explained by the model (*R*^2^ = 0.08, *F*_(1,57)_ = 5.03, *P* = 0.015) (cf. **Figure 1**; for further detail, view **SI and Figure S1**). After Bonferroni-correction, we observed a trend toward significance for this association (*P* = 0.06). Salivary oxytocin levels were significantly increased after intranasal OXT administration relative to intranasal PLC administration, which we reported in (27). However, CTQ sum scores did not predict the modulatory effect of OXT on stress-specific fearful recognition ratings across all four emotion intensity levels (all *P*s > 0.05; for more detail, view **SI**). Moreover, CTQ sum scores did not predict fearful recognition ratings for all emotion intensities during trials in which subjects were exposed to the non-social control odor raspberry under PLC (all *P*s > 0.05; for more detail, see **SI**).

Correlation analyses did not yield significant associations between CTQ sum scores and post fMRI pleasantness, intensity, and familiarity ratings of either social or non-social odor stimuli (all *P*s ≥ 0.05; for further detail, view **SI**). Thus, CM did not influence the perception of odor quality.

### fMRI Results

**Figure 2:**
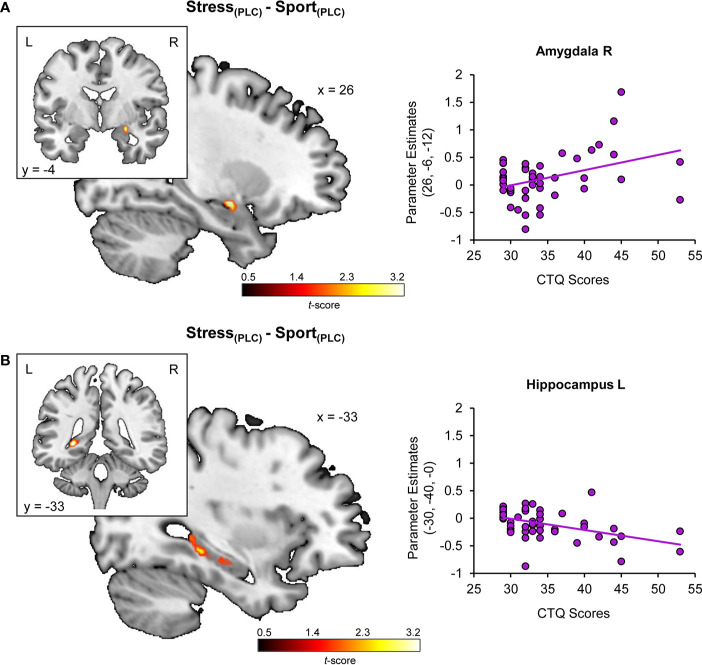
Childhood maltreatment and the impact of chemosensory stress signals on **(A)** amygdala and **(B)** hippocampus reactivity. Childhood Trauma Questionnaire (CTQ) scores are associated with a stress-specific amygdala hyperreactivity and hippocampal deactivation. CTQ, Childhood Trauma Questionnaire; PLC, placebo; L, left hemisphere; R, right hemisphere.

**Figure 3:**
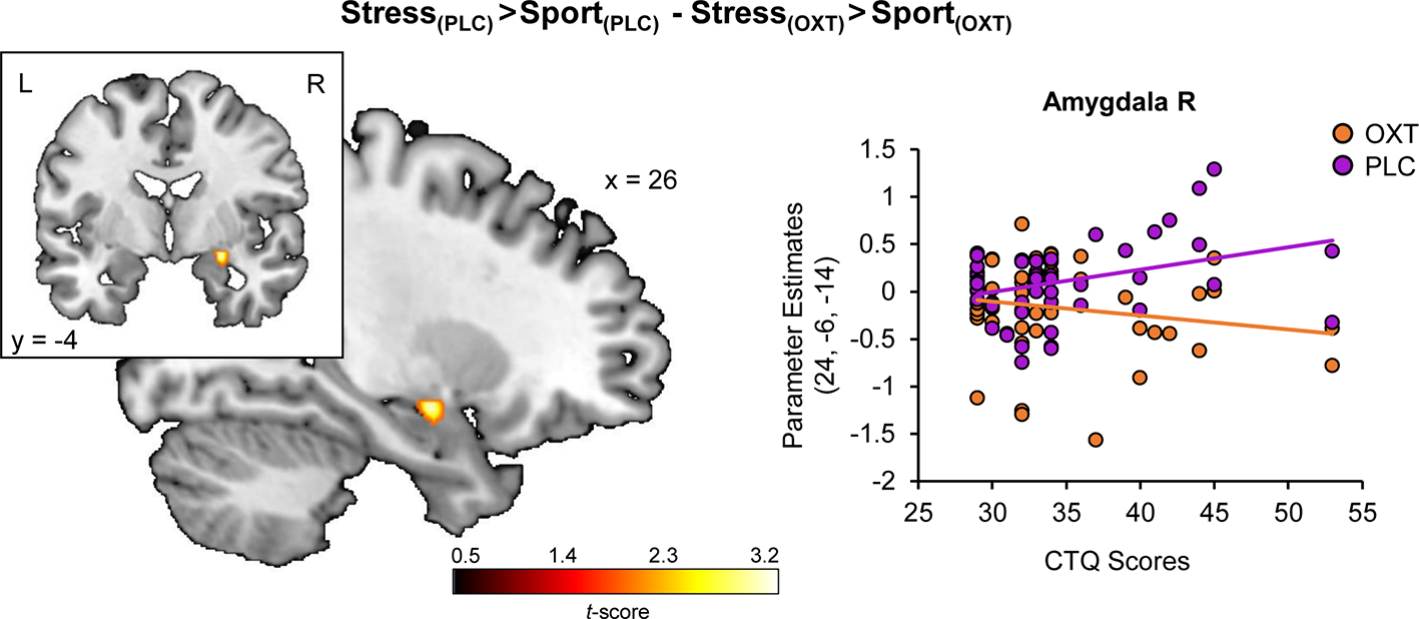
Childhood maltreatment and the modulatory effect of oxytocin on stress-specific amygdala reactivity. Childhood Trauma Questionnaire (CTQ) scores are associated with a stress-specific dampening effect of oxytocin in the amygdala. OXT, oxytocin; PLC, placebo; L, left hemisphere; R, right hemisphere.

Regression analyses yielded a positive association of CTQ sum scores and stress-specific right amygdala hyperreactivity (peak MNI coordinates x, y, z: 26, −6, −12; *t*_(48)_ = 3.51, *P*_FWE_ = 0.015) (cf. **Figure 2A**) and a negative association of CTQ sum scores and stress-specific left hippocampal hyporeactivity (−30, −40, 0; *t*_(48)_ = 3.96, *P*_FWE_ = 0.017) (cf. **Figure 2B**) under PLC (for further detail, view **Figure S2**). Furthermore, CTQ scores were positively associated with the stress-specific effect of OXT in the right amygdala (24, −6, −14; *t*_(48)_ = 3.41, *P*_FWE_ = 0.038) (cf. **Figure 3**). Stress-associated increases in amygdala reactivity suggest CM may induce hypervigilance to chemosensory threat cues in the present sample. Moreover, stress-specific attenuating effects of OXT in the amygdala appear to be more pronounced in participants with increasing levels of CM exposure.

### Connectivity Results

**Figure 4:**
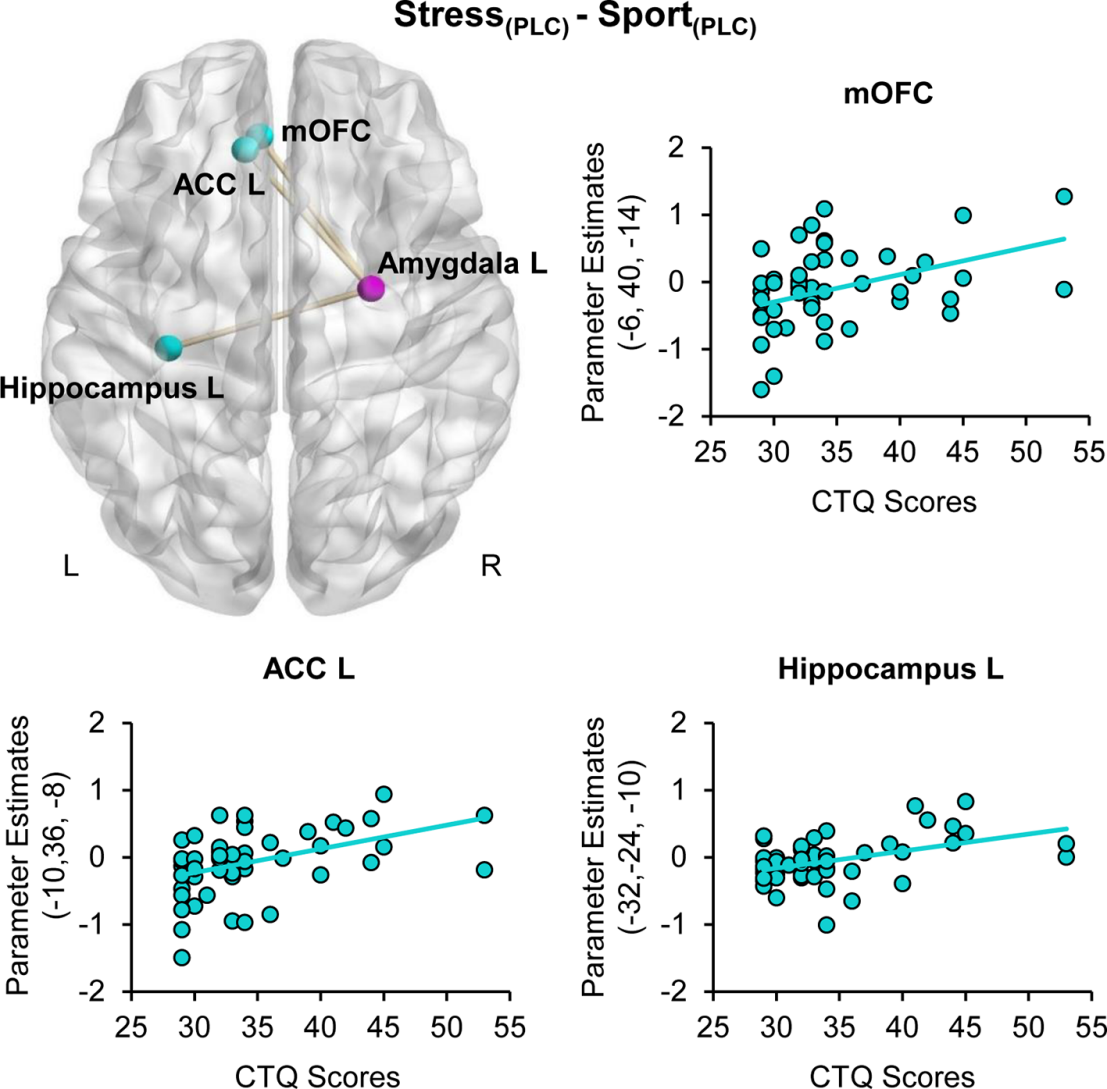
Childhood maltreatment and stress-specific functional connectivity. Childhood Trauma Questionnaire (CTQ) CTQ scores were associated with a heightened functional connectivity between the amygdala (purple sphere) and the medial orbitofrontal cortex (mOFC), the anterior cingulate cortex (ACC) and the hippocampus (blue spheres) when subjects were exposed to stress sweat. ACC, anterior cingulate cortex; CTQ, Childhood Trauma Questionnaire; mOFC, medial orbitofrontal cortex; PLC, placebo; L, left hemisphere; R, right hemisphere.

For the right amygdala seed region (26, −6, −12), the gPPI analysis revealed a stress-specific positive association of CTQ scores and functional coupling with the left mOFC (peak MNI coordinates x, y, z: −6, 40, −14; *t*_(48)_ =4.09, *P*_FWE_ = 0.019, ACC (−10, 36, −8; *t*_(48)_ = 4.08, *P*_FWE_ = 0.039) and hippocampus (−32, −24, −10; *t*_(48)_ =3.87, *P*_FWE_ =0.046) under PLC (cf. **Figure 4**). Furthermore, we observed a positive association of CTQ scores with OXT effects for the functional coupling between the right amygdala seed region (26, −6, −12) and the left mOFC (−2, 28, −12; *t*_(48)_ = 4.41, *P*_FWE_ = 0.008) in the stress relative to the sport condition. There were no CTQ-associated changes in FC for the hippocampus as a seed region. The CTQ-associated increase in FC may reflect an inefficient top-down regulation of the amygdala *via* the ACC and the mOFC. Administration of intranasal OXT appear to reinstate the frontolimbic regulatory mechanism.

### Mediation and Moderation Effects

We did not detect significant mediation or moderation effects for any covariate. All 95% confidence intervals of indirect effects overlapped with zero and all interaction terms between CM and moderation variables were non-significant (all *P*s > 0.05). Thus, the observed modulatory effect of CM on the behavioral and neural levels were not significantly moderated or mediated by sociodemographic factors, depression or anxiety levels.

### Effect of Maltreatment Type

Multiple regression analyses with the five CTQ subscales as predictors revealed an association of emotional neglect (*β* = −0.36, *P* = 0.04) and emotional abuse (*β* = 0.54, *P* = 0.004) with the chemosensory induced bias in the recognition of high fearful faces under PLC (*β* = 0.31, *P* = 0.016). Stress-specific amygdala hyperreactivity was associated with emotional neglect (*β* = 0.53 P = 0.002) and physical neglect (*β* = 0.29, *P* = 0.022). Entering all five subscales into the model did not reveal an association of hippocampal hypoactivation with a specific subscale (all *P*s > 0.05). Stress-specific FC between the amygdala and the hippocampus was associated with emotional abuse (*β* = 0.43, *P* = 0.019). Multiple regression analysis revealed no association of amygdala-ACC FC with a specific CM subscale. Stress-specific FC between the amygdala and the mOFC was associated with sexual abuse (*β* = 0.29, *P* = 0.043). We observed no significant association between the stress-reducing effects of OXT and specific CM subtypes. Correlation analysis revealed that the subscale emotional neglect highly correlated with the subscale emotional abuse (*r* = 0.64, *P* < 0.001) and moderately correlated with physical neglect (*r* = 0.3, *P* = 0.023).

## Discussion

In the present study, we primarily examined the modulatory impact of CM on the processing of chemosensory threat signals in axillary sweat. Given the long-term consequences of CM on the oxytocinergic system, our secondary aim was to investigate whether CM affects the stress-attenuating effects of OXT in this context. As expected, a secondary analysis of our recent study (27) revealed a modulating role for CM in the olfactory domain of social threat communication. CM was associated with increased amygdala reactivity, decreased hippocampal activation and increased FC between the amygdala and the hippocampus, ACC and mOFC during exposure to threat-associated olfactory signals. This neural response pattern was paralleled by a threat-related increase in the recognition of high fearful faces. Furthermore, in line with our second hypothesis, we found that CM moderated the effects of OXT on threat-related processing of these olfactory signals. The observed response pattern was not moderated or mediated by sociodemographic factors, current depression or trait anxiety levels. Here, we extend previous evidence of a phenotypic hypervigilance in adults with a history of CM (4) to the olfactory domain, highlighting an underexplored vulnerability pathway to psychopathology in those affected.

Our finding of CM-associated amygdala hyperreactivity to social olfactory threat cues is directly in line with frequently reported elevated amygdala responses to threatening faces in individuals with a history of CM (7–9). Moreover, converging evidence demonstrates changes in frontolimbic FC following CM both at rest (75) and during emotional face processing tasks (10, 11, 59). While this response pattern may reflect a mechanism mediating resilience when measured at rest, increased task-based FC between the amygdala, the ACC and the OFC has been linked to an inefficient regulatory system in adults following CM (56). It is well established that the amygdala, hippocampus, ACC, and PFC are central to efficient threat and fear regulation (76, 77). Both the ACC and the mOFC exert top-down control on limbic and endocrine systems through mechanisms such as attentional control and contextual processing (16–18, 77). Our findings suggest that threat-associated amygdala activation prompted individuals with a history of CM to up-regulate activations of cognitive control regions. In healthy adults, increased FC between the amygdala and the OFC as well as the ACC was associated with threat-induced anxiety (78) and in trauma-exposed adolescents increased amygdala-ACC connectivity was paralleled by a reduced ability to regulate emotional conflict (79). In the current sample, elevated amygdala reactivity and concomitantly increased frontolimbic FC might reflect hypervigilance to the threatening properties of the olfactory signals used in the fMRI paradigm. Further, our data support the notion that CM is associated with long-term downstream perturbations of frontolimbic emotion circuits (1, 4). Previous data show hippocampal hypoactivation both following psychosocial stress induction (80) and in response to masked fearful faces (81) in individuals with a history of CM. By contrast, other studies found increased activation of the hippocampus in response to threatening faces (82, 83). These conflicting findings may arise from variations in the operationalization of CM, time between trauma exposure and data collection and psychiatric comorbidities. However, accumulating evidence suggests CM-related hippocampal deactivation in response to emotional faces may represent a mechanism of resilience (57). The hippocampus and the amygdala are highly susceptible to adaptions following early life stress (4) and subtle interactions between these structures are central for forming representations of emotional significance and contextually modulating physiological threat responses (77, 84). Furthermore, amygdala-hippocampal FC is crucially involved in the regulation of the hypothalamic-pituitary-adrenal (HPA) (85) axis and has been shown to predict the capacity of the HPA axis to restore homeostasis of the stress response after perturbations (86). Along this line, there is evidence showing that upon induction of psychosocial stress, deactivation of the hippocampus plays an essential role in initiating a stress response (87). Notably, recent evidence demonstrates that CM-related differences in amygdala-hippocampus FC in response to threatening facial stimuli are linked to adult adaptive functioning (59). Thus, we propose that enhanced recruitment of the amygdala-hippocampus complex during exposure to social olfactory threat cues might reflect a compensatory mechanism for inefficient frontolimbic circuitry in individuals with a history of CM. Resilient individuals with a history of CM may exhibit an enhanced capacity for contextualizing social olfactory threat signals due to an amygdala-dependent increased presentation of these cues in the hippocampus (77). Enhanced encoding of these signals may allow resilient individuals with a history of CM to adaptively refine physiological stress responses in a safe context.

In the current sample, CM-related neural responses to social olfactory threat signals were paralleled by increased recognition of fearful faces. This shows that the cross-modal sensory integration of visual and olfactory threat cues is modulated by a history of CM. Previous findings revealed that effective olfactory-visual emotion integration results in biased detection of fear that is accompanied by enhanced amygdala responsiveness and increased functional connectivity between the amygdala and the OFC (88). Moreover, the ACC and the OFC have been suggested to be part of a network that initiates increased sensory responses during cross-modal sensory integration of fear (89). Thus, given their hypervigilant sensory profile, individuals with a history of CM may exhibit increased evaluation of social olfactory threat cues that leads to biased emotion detection. (7). Enhanced cross-modal fear detection during the exposure of social olfactory threats may represent an adaptive mechanism, by which individuals accentuate their automatic response in a threatening environment (4).

Burgeoning evidence implicates the oxytocinergic system in CM (1). Mechanistically, a stronger effect of OXT on threat-specific amygdala activation in individuals with a history of CM appears to be rooted in a reinstated top-down regulatory function of the mOFC over the amygdala, thereby emulating a more normative response to social olfactory threat cues in individuals with a history of CM (16–19, 76). Evidence corroborating this interpretation comes from studies demonstrating that OXT reduces threat hypersensitivity in women with Borderline Personality Disorder (90, 91) which is frequently associated with CM. Likewise, OXT enhanced the stress-buffering effects of social support in women with more severe CM exposure (51). The oxytocinergic system is highly sensitive to the adverse effects of CM, with most studies reporting decreased levels of peripheral and central OXT in a dose-dependent manner following CM exposure (44–46). These findings could reflect a downregulation of the OXT system and increased OXT sensitivity in individuals with higher levels of CM exposure. However, previous studies also reported diminished stress-attenuating (92) or prosocial (93) effects of OXT in individuals who have experienced CM. Thus, the moderating role of CM on OXT effects is also evident in the olfactory domain, but the direction of this moderation seems to vary depending on baseline differences and sample characteristics.

The stress-specific chemosensory effects were predominantly associated with emotional and physical neglect as well as emotional abuse subscales. However, given the high intercorrelation of the subscales, these results need to be interpreted carefully. Importantly, our moderation and median analyses revealed that CM-associated symptoms, such as depression and anxiety, did not significantly influence the observed pattern of results. Furthermore, recent findings of CM-related structural and functional alterations (37) as well as aberrant responses to non-social olfactory threat cues (94) corroborate the notion of an etiological olfactory pathway to psychopathology in individuals with a history of CM. Consistent with this idea, enhanced amygdala reactivity to threatening facial stimuli has been found to mediate the link between CM and the development of adult anxiety disorders and PTSD (14, 15). Thus, future work is warranted to examine whether the observed alterations of the social olfactory pathway precipitate a latent vulnerability to later psychopathology in the context of CM.

There are a number of limitations in this study that need to be addressed in future research. First, the retrospective and self-report assessment of CM may be subject to misreporting of CM. While we did thoroughly control for current anxiety and depression levels, which may provoke a negative recall bias (95), we cannot exclude that a recall-related underreporting of CM in the present healthy sample may have influenced our results (96). Second, we were not able to ascertain whether the observed alterations in olfactory processing were associated with specific types of maltreatment due to the interrelatedness of CM types in the present sample. Given that various forms of CM frequently co-occur (2), future studies employing a longitudinal design are needed to probe the associations between specific forms of CM, neural responses to olfactory threat cues, and psychopathology. Third, while the study used a well-controlled healthy sample, subjects of the study exhibited mild CM, limiting the interpretation of the findings to the context of less severe forms of CM. However, given the robust finding of dose-dependent effects of CM, we speculate that a neural threat response to olfactory signals may also be observed in individuals with a history of severe CM exposure. Fourth, here we report that CM is associated with altered responses to social olfactory stress cues compared to sport-related social olfactory cues. However, in contrast to the difference scores, we did not observe a significant association between CM and parameter estimates of the amygdala and hippocampus responses to stress and sport odor cues compared to baseline (cf. SI). Thus, it is conceivable that a differential response to sport odor cues contributed to the observed CM-associated changes. Future studies should include additional non-stress-related social control conditions in their design to further investigate the specificity of stress-related responses in subjects with a history of CM. Finally, while the fMRI analysis did not include a correction for small frame-to-frame head movements, an additional control analysis demonstrated that subjects in the present sample exhibited no critical head movements during scanning.

In conclusion, we extend prior findings of a phenotypic hypervigilance to social threat signals in individuals with a history of CM to the domain of social olfactory signals. We propose that CM disrupts the neural circuitry of threat detection by weakening top-down regulatory systems. Increased intralimbic connectivity may reflect an effective compensatory mechanism in resilient individuals. Furthermore, CM moderates the effects of OXT on the processing of chemosensory stress signals. The current study highlights a potential vulnerability pathway in individuals with a history of CM that needs to be addressed in future work.

## Data Availability Statement

The raw data supporting the conclusions of this article will be made available by the authors, without undue reservation.

## Ethics Statement

The studies involving human participants were reviewed and approved by Ethikkommission an der medizinischen Fakultät der Rheinischen Friedrich-Wilhelms-Universität Bonn. The patients/participants provided their written informed consent to participate in this study.

## Author Contributions

AM and DS designed the experiments. AM and LH-L conducted the experiments. AM, LH-L, and DS analyzed the data. All authors wrote the manuscript. All authors contributed to the article and approved the submitted version.

## Funding

DS is supported by an Else-Kröner-Fresenius-Stiftung grant (2017_A35).

## Conflict of Interest

The authors declare that the research was conducted in the absence of any commercial or financial relationships that could be construed as a potential conflict of interest.

## References

[1] Nemeroff CB Paradise Lost: The Neurobiological and Clinical Consequences of Child Abuse and Neglect. Neuron (2016) 89(5):892–909. 10.1016/j.neuron.2016.01.019 26938439

[2] Green JG McLaughlin KA Berglund PA Gruber MJ Sampson NA Zaslavsky AM Childhood adversities and adult psychiatric disorders in the national comorbidity survey replication I: associations with first onset of DSM-IV disorders. Arch Gen Psychiat (2010) 67(2):113–23. 10.1001/archgenpsychiatry.2009.186 PMC2822662 20124111

[3] Berens AE Jensen SKG Nelson CA,3 Biological embedding of childhood adversity: from physiological mechanisms to clinical implications. BMC Med (2017) 15(1):135. 10.1186/s12916-017-0895-4 28724431 PMC5518144

[4] Teicher MH Samson JA Anderson CM Ohashi K The effects of childhood maltreatment on brain structure, function and connectivity. Nat Rev Neurosci (2016) 17(10):652–66. 10.1038/nrn.2016.111 27640984

[5] Herzog JI Schmahl C Adverse Childhood Experiences and the Consequences on Neurobiological, Psychosocial, and Somatic Conditions Across the Lifespan. Front Psychiatry (2018) 9:420:420. 10.3389/fpsyt.2018.00420 30233435 PMC6131660

[6] Maier A Gieling C Heinen-Ludwig L Stefan V Schultz J Gunturkun O Association of Childhood Maltreatment With Interpersonal Distance and Social Touch Preferences in Adulthood. Am J Psychiatry (2020) 177(1):37–46. 10.1176/appi.ajp.2019.19020212 31416339

[7] McCrory EJ De Brito SA Sebastian CL Mechelli A Bird G Kelly PA Heightened neural reactivity to threat in child victims of family violence. Curr Biol (2011) 21(23):R947–8. 10.1016/j.cub.2011.10.015 22153160

[8] Zhu J Lowen SB Anderson CM Ohashi K Khan A Teicher MH Association of Prepubertal and Postpubertal Exposure to Childhood Maltreatment With Adult Amygdala Function. JAMA Psychiatry (2019) 76(8):843–53. 10.1001/jamapsychiatry.2019.0931 PMC6596335 31241756

[9] Dannlowski U Stuhrmann A Beutelmann V Zwanzger P Lenzen T Grotegerd D Limbic scars: long-term consequences of childhood maltreatment revealed by functional and structural magnetic resonance imaging. Biol Psychiatry (2012) 71(4):286–93. 10.1016/j.biopsych.2011.10.021 22112927

[10] Fonzo GA Flagan TM Sullivan S Allard CB Grimes EM Simmons AN Neural functional and structural correlates of childhood maltreatment in women with intimate-partner violence-related posttraumatic stress disorder. Psychiatry Res (2013) 211(2):93–103. 10.1016/j.pscychresns.2012.08.006 23154098 PMC3570713

[11] Jedd K Hunt RH Cicchetti D Hunt E Cowell RA Rogosch FA Long-term consequences of childhood maltreatment: Altered amygdala functional connectivity. Dev Psychopathol (2015) 27(4 Pt 2):1577–89. 10.1017/S0954579415000954 PMC4635964 26535945

[12] Feinstein JS Adolphs R Damasio A Tranel D The human amygdala and the induction and experience of fear. Curr Biol (2011) 21(1):34–8. 10.1016/j.cub.2010.11.042 PMC3030206 21167712

[13] McTeague LM Rosenberg BM Lopez JW Carreon DM Huemer J Jiang Y Identification of Common Neural Circuit Disruptions in Emotional Processing Across Psychiatric Disorders. Am J Psychiatry (2020) 177(5):411–21. 10.1176/appi.ajp.2019.18111271 PMC7280468 31964160

[14] Fonzo GA Ramsawh HJ Flagan TM Simmons AN Sullivan SG Allard CB Early life stress and the anxious brain: evidence for a neural mechanism linking childhood emotional maltreatment to anxiety in adulthood. Psychol Med (2016) 46(5):1037–54. 10.1017/S0033291715002603 PMC4795156 26670947

[15] Lanius RA Bluhm R Lanius U Pain C A review of neuroimaging studies in PTSD: heterogeneity of response to symptom provocation. J Psychiatr Res (2006) 40(8):709–29. 10.1016/j.jpsychires.2005.07.007 16214172

[16] Ghashghaei HT Hilgetag CC Barbas H Sequence of information processing for emotions based on the anatomic dialogue between prefrontal cortex and amygdala. Neuroimage (2007) 34(3):905–23. 10.1016/j.neuroimage.2006.09.046 PMC2045074 17126037

[17] Etkin A Egner T Kalisch R Emotional processing in anterior cingulate and medial prefrontal cortex. Trends Cognit Sci (2011) 15(2):85–93. 10.1016/j.tics.2010.11.004 21167765 PMC3035157

[18] Rule RR Shimamura AP Knight RT Orbitofrontal cortex and dynamic filtering of emotional stimuli. Cognit Affect Behav Neurosci (2002) 2(3):264–70. 10.3758/cabn.2.3.264 12775190

[19] Banks SJ Eddy KT Angstadt M Nathan PJ Phan KL Amygdala-frontal connectivity during emotion regulation. Soc Cognit Effect Neurosci (2007) 2(4):303–12. 10.1093/scan/nsm029 PMC2566753 18985136

[20] Stevenson RJ An initial evaluation of the functions of human olfaction. Chem Senses (2010) 35(1):3–20. 10.1093/chemse/bjp083 19942579

[21] Sarafoleanu C Mella C Georgescu M Perederco C The importance of the olfactory sense in the human behavior and evolution. J Med Life (2009) 2(2):196–8. PMC3018978 20108540

[22] Lubke KT Pause BM Always follow your nose: the functional significance of social chemosignals in human reproduction and survival. Horm Behav (2015) 68:134–44. 10.1016/j.yhbeh.2014.10.001 25637403

[23] de Groot JH Smeets MA Kaldewaij A Duijndam MJ Semin GR Chemosignals communicate human emotions. Psychol Sci (2012) 23(11):1417–24. 10.1177/0956797612445317 23019141

[24] Mutic S Brunner YF Rodriguez-Raecke R Wiesmann M Freiherr J Chemosensory danger detection in the human brain: Body odor communicating aggression modulates limbic system activation. Neuropsychologia (2017) 99:187–98. 10.1016/j.neuropsychologia.2017.02.018 28254652

[25] Doucet S Soussignan R Sagot P Schaal B The secretion of areolar (Montgomery’s) glands from lactating women elicits selective, unconditional responses in neonates. PloS One (2009) 4(10):e7579. 10.1371/journal.pone.0007579 19851461 PMC2761488

[26] Ferdenzi C Licon C Bensafi M Detection of sickness in conspecifics using olfactory and visual cues. PNAS (2017) 114(24):6157–9. 10.1073/pnas.1707139114 PMC5474810 28584131

[27] Maier A Scheele D Spengler FB Menba T Mohr F Gunturkun O Oxytocin reduces a chemosensory-induced stress bias in social perception. Neuropsychopharmacology (2019) 44(2):281–8. 10.1038/s41386-018-0063-3 PMC6300531 29703998

[28] Wudarczyk OA Kohn N Bergs R Goerlich KS Gur RE Turetsky B Chemosensory anxiety cues enhance the perception of fearful faces - An fMRI study. Neuroimage (2016) 143:214–22. 10.1016/j.neuroimage.2016.09.002 27592811

[29] Mujica-Parodi LR Strey HH Frederick B Savoy R Cox D Botanov Y Chemosensory cues to conspecific emotional stress activate amygdala in humans. PloS One (2009) 4(7):e6415. 10.1371/journal.pone.0006415 19641623 PMC2713432

[30] Prehn-Kristensen A Wiesner C Bergmann TO Wolff S Jansen O Mehdorn HM Induction of empathy by the smell of anxiety. PloS One (2009) 4(6):e5987. 10.1371/journal.pone.0005987 19551135 PMC2695008

[31] Pause BM Adolph D Prehn-Kristensen A Ferstl R Startle response potentiation to chemosensory anxiety signals in socially anxious individuals. Int J Psychophysiol (2009) 74(2):88–92. 10.1016/j.ijpsycho.2009.07.008 19666058

[32] Wintermann GB Donix M Joraschky P Gerber J Petrowski K Altered olfactory processing of stress-related body odors and artificial odors in patients with panic disorder. PloS One (2013) 8(9):e74655. 10.1371/journal.pone.0074655 24086358 PMC3782473

[33] Gottfried JA Central mechanisms of odour object perception. Nat Rev Neurosci (2010) 11(9):628–41. 10.1038/nrn2883 PMC3722866 20700142

[34] Soudry Y Lemogne C Malinvaud D Consoli SM Bonfils P Olfactory system and emotion: common substrates. Eur Ann Otorhinolaryngol Head Neck Dis (2011) 128(1):18–23. 10.1016/j.anorl.2010.09.007 21227767

[35] Rolls ET Kringelbach ML de Araujo IE Different representations of pleasant and unpleasant odours in the human brain. Eur J Neurosci (2003) 18(3):695–703. 10.1046/j.1460-9568.2003.02779.x 12911766

[36] Croy I Schellong J Gerber J Joraschky P Iannilli E Hummel T Women with a history of childhood maltreatment exhibit more activation in association areas following non-traumatic olfactory stimuli: a fMRI study. PloS One (2010) 5(2):e9362. 10.1371/journal.pone.0009362 20179758 PMC2825260

[37] Croy I Negoias S Symmank A Schellong J Joraschky P Hummel T Reduced olfactory bulb volume in adults with a history of childhood maltreatment. Chem Senses (2013) 38(8):679–84. 10.1093/chemse/bjt037 24051351

[38] Dileo JF Brewer WJ Hopwood M Anderson V Creamer M Olfactory identification dysfunction, aggression and impulsivity in war veterans with post-traumatic stress disorder. Psychol Med (2008) 38(4):523–31. 10.1017/S0033291707001456 17903334

[39] Wilkerson AK Uhde TW Leslie K Freeman WC LaRowe SD Schumann A Paradoxical olfactory function in combat veterans: The role of PTSD and odor factors. Mil Psychol (2018) 30(2):120–30. 10.1080/08995605.2018.1425063 PMC6132269 30220788

[40] Cortese BM Schumann AY Howell AN McConnell PA Yang QX Uhde TW Preliminary evidence for differential olfactory and trigeminal processing in combat veterans with and without PTSD. NeuroImage Clin (2018) 17:378–87. 10.1016/j.nicl.2017.09.018 PMC5683811 29159050

[41] Meyer-Lindenberg A Domes G Kirsch P Heinrichs M Oxytocin and vasopressin in the human brain: social neuropeptides for translational medicine. Nat Rev Neurosci (2011) 12(9):524–38. 10.1038/nrn3044 21852800

[42] Pena CJ Kronman HG Walker DM Cates HM Bagot RC Purushothaman I Early life stress confers lifelong stress susceptibility in mice via ventral tegmental area OTX2. Science (2017) 356(6343):1185–8. 10.1126/science.aan4491 PMC5539403 28619944

[43] Francis DD Champagne FC Meaney MJ Variations in maternal behaviour are associated with differences in oxytocin receptor levels in the rat. J Neuroendocrinol (2000) 12(12):1145–8. 10.1046/j.1365-2826.2000.00599.x 11106970

[44] Kojima S Stewart RA Demas GE Alberts JR Maternal contact differentially modulates central and peripheral oxytocin in rat pups during a brief regime of mother-pup interaction that induces a filial huddling preference. J Neuroendocrinol (2012) 24(5):831–40. 10.1111/j.1365-2826.2012.02280.x PMC4060530 22260655

[45] Opacka-Juffry J Mohiyeddini C Experience of stress in childhood negatively correlates with plasma oxytocin concentration in adult men. Stress (2012) 15(1):1–10. 10.3109/10253890.2011.560309 21682649

[46] Heim C Young LJ Newport DJ Mletzko T Miller AH Nemeroff CB Lower CSF oxytocin concentrations in women with a history of childhood abuse. Mol Psychiatry (2009) 14(10):954–8. 10.1038/mp.2008.112 18957940

[47] Mizuki R Fujiwara T Association of oxytocin level and less severe forms of childhood maltreatment history among healthy Japanese adults involved with child care. Front Behav Neurosci (2015) 9:138:138. 10.3389/fnbeh.2015.00138 26157369 PMC4477143

[48] Kirschbaum C Pirke KM Hellhammer DH The Trier Social Stress Test - a Tool for Investigating Psychobiological Stress Responses in a Laboratory Setting. Neuropsychobiology (1993) 28(1-2):76–81. 10.1159/000119004 8255414

[49] Pierrehumbert B Torrisi R Laufer D Halfon O Ansermet F Beck Popovic M Oxytocin response to an experimental psychosocial challenge in adults exposed to traumatic experiences during childhood or adolescence. Neurosci (2010) 166(1):168–77. 10.1016/j.neuroscience.2009.12.016 20018229

[50] Heinrichs M Baumgartner T Kirschbaum C Ehlert U Social support and oxytocin interact to suppress cortisol and subjective responses to psychosocial stress. Biol Psychiatry (2003) 54(12):1389–98. 10.1016/s0006-3223(03)00465-7 14675803

[51] Riem MME Kunst LE Bekker MHJ Fallon M Kupper N Intranasal oxytocin enhances stress-protective effects of social support in women with negative childhood experiences during a virtual Trier Social Stress Test. Psychoneuroendocrinology (2020) 111:104482. 10.1016/j.psyneuen.2019.104482 31677411

[52] Scheele D Kendrick KM Khouri C Kretzer E Schlapfer TE Stoffel-Wagner B An oxytocin-induced facilitation of neural and emotional responses to social touch correlates inversely with autism traits. Neuropsychopharmacology (2014) 39(9):2078–85. 10.1038/npp.2014.78 PMC4104346 24694924

[53] Kreuder AK Scheele D Wassermann L Wollseifer M Stoffel-Wagner B Lee MR How the brain codes intimacy: The neurobiological substrates of romantic touch. Hum Brain Mapp (2017) 38(9):4525–34. 10.1002/hbm.23679 PMC6867116 28580708

[54] Bakermans-Kranenburg MJ van Ijzendoorn MH Riem MM Tops M Alink LR Oxytocin decreases handgrip force in reaction to infant crying in females without harsh parenting experiences. Soc Cognit Affect Neurosci (2012) 7(8):951–7. 10.1093/scan/nsr067 PMC3501699 22037689

[55] Gilbert R Widom CS Browne K Fergusson D Webb E Janson S Burden and consequences of child maltreatment in high-income countries. Lancet (2009) 373(9657):68–81. 10.1016/S0140-6736(08)61706-7 19056114

[56] Vachon DD Krueger RF Rogosch FA Cicchetti D Assessment of the Harmful Psychiatric and Behavioral Effects of Different Forms of Child Maltreatment. JAMA Psychiatry (2015) 72(11):1135–42. 10.1001/jamapsychiatry.2015.1792 PMC4699442 26465073

[57] Moreno-Lopez L Ioannidis K Askelund AD Smith AJ Schueler K van Harmelen AL The Resilient Emotional Brain: A Scoping Review of the Medial Prefrontal Cortex and Limbic Structure and Function in Resilient Adults With a History of Childhood Maltreatment. Biol Psychiatry Cognit Neurosci Neuroimaging (2019) 5(4):392–402. 10.1016/j.bpsc.2019.12.008 32115373

[58] Ioannidis K Askelund AD Kievit RA van Harmelen AL The complex neurobiology of resilient functioning after childhood maltreatment. BMC Med (2020) 18(1):32. 10.1186/s12916-020-1490-7 32050974 PMC7017563

[59] Demers LA McKenzie KJ Hunt RH Cicchetti D Cowell RA Rogosch FA Separable Effects of Childhood Maltreatment and Adult Adaptive Functioning on Amygdala Connectivity During Emotion Processing. Biol Psychiatry Cognit Neurosci Neuroimaging (2018) 3(2):116–24. 10.1016/j.bpsc.2017.08.010 PMC5851478 29529406

[60] Bernstein DP Stein JA Newcomb MD Walker E Pogge D Ahluvalia T Development and validation of a brief screening version of the Childhood Trauma Questionnaire. Child Abuse Negl (2003) 27(2):169–90. 10.1016/s0145-2134(02)00541-0 12615092

[61] Sheehan DV Lecrubier Y Sheehan KH Amorim P Janavs J Weiller E The Mini-International Neuropsychiatric Interview (M.I.N.I.): the development and validation of a structured diagnostic psychiatric interview for DSM-IV and ICD-10. J Clin Psychiatry (1998) 59(Suppl 20):22–33;quiz 4-57. 9881538

[62] Bernstein DP Fink L Handelsman L Fotte J M.Lovejoy K Wenzel K Initial reliability and validity of a new retrospective measure of child abuse and neglect. Am J Psychiatry (1994) 151(8):1132–6. 10.1176/ajp.151.8.1132 8037246

[63] Beck AT Steer RA Brown GK Manual for the Beck Depression Inventory-II. San Antonio TX: Psychological Corporation (1996).

[64] Spielberger CD Gorsuch RL Lushene RE Manual for the State-Trait Anxiety Inventory. Palo Alto, CA: Consulting Psychologists Press (1970).

[65] Baron-Cohen S Wheelwright S Skinner R Martin J Clubley E The Autism-Spectrum Quotient (AQ): Evidence from Asperger syndrome/high-functioning autism, males and females, scientists and mathematicians. J Autism Dev Disord (2001) 31(1):5–17. 10.1023/A:1005653411471 11439754

[66] Guastella AJ Hickie IB McGuinness MM Otis M Woods EA Disinger HM Recommendations for the standardisation of oxytocin nasal administration and guidelines for its reporting in human research. Psychoneuroendocrinology (2013) 38(5):612–25. 10.1016/j.psyneuen.2012.11.019 23265311

[67] Hummel T Sekinger B Wolf SR Pauli E Kobal G ‘Sniffin’ Sticks’: Olfactory performance assessed by the combined testing of odor identification, odor discrimination and olfactory threshold. Chem Senses (1997) 22(1):39–52. 10.1093/chemse/22.1.39 9056084

[68] Kasper L Bollmann S Diaconescu AO Hutton C Heinzle J Iglesias S The PhysIO Toolbox for Modeling Physiological Noise in fMRI Data. J Neurosci Methods (2017) 276:56–72. 10.1016/j.jneumeth.2016.10.019 27832957

[69] McLaren DG Ries ML Xu G Johnson SC A generalized form of context-dependent psychophysiological interactions (gPPI): a comparison to standard approaches. NeuroImage (2012) 61(4):1277–86. 10.1016/j.neuroimage.2012.03.068 PMC3376181 22484411

[70] Power JD Barnes KA Snyder AZ Schlaggar BL Petersen SE Spurious but systematic correlations in functional connectivity MRI networks arise from subject motion. NeuroImage (2012) 59(3):2142–54. 10.1016/j.neuroimage.2011.10.018 PMC3254728 22019881

[71] Ciric R Wolf DH Power JD Roalf DR Baum GL Ruparel K Benchmarking of participant-level confound regression strategies for the control of motion artifact in studies of functional connectivity. NeuroImage (2017) 154:174–87. 10.1016/j.neuroimage.2017.03.020 PMC5483393 28302591

[72] Siegel JS Power JD Dubis JW Vogel AC Church JA Schlaggar BL Statistical improvements in functional magnetic resonance imaging analyses produced by censoring high-motion data points. Hum Brain Mapp (2014) 35:1981–96. 10.1002/hbm.22307 PMC3895106 23861343

[73] Hayes AF Introduction to mediation, moderation, and conditional process analysis: A regression based approach. New York, NY: Guilford Press (2013).

[74] Baron RM Kenny DA The moderator-mediator variable distinction in social psychological research: conceptual, strategic, and statistical considerations. J Pers Soc Psychol (1986) 51(6):1173–82. 10.1037/0022-3514.51.6.1173 3806354

[75] Herringa RJ Birn RM Ruttle PL Burghy CA Stodola DE Davidson RJ Childhood maltreatment is associated with altered fear circuitry and increased internalizing symptoms by late adolescence. PNAS (2013) 110(47):19119–24. 10.1073/pnas.1310766110 PMC3839755 24191026

[76] Shin LM Liberzon I The neurocircuitry of fear, stress, and anxiety disorders. Neuropsychopharmacology (2010) 35(1):169–91. 10.1038/npp.2009.83 PMC3055419 19625997

[77] Maren S Phan KL Liberzon I The contextual brain: implications for fear conditioning, extinction and psychopathology. Nat Rev Neurosci (2013) 14(6):417–28. 10.1038/nrn3492 PMC5072129 23635870

[78] Gold AL Morey RA McCarthy G Amygdala-prefrontal cortex functional connectivity during threat-induced anxiety and goal distraction. Biol Psychiatry (2015) 77(4):394–403. 10.1016/j.biopsych.2014.03.030 24882566 PMC4349396

[79] Marusak HA Martin KR Etkin A Thomason ME Childhood trauma exposure disrupts the automatic regulation of emotional processing. Neuropsychopharmacology (2015) 40(5):1250–8. 10.1038/npp.2014.311 PMC4367470 25413183

[80] Grimm S Pestke K Feeser M Aust S Weigand A Wang J Early life stress modulates oxytocin effects on limbic system during acute psychosocial stress. Soc Cognit Affect Neurosci (2014) 9(11):1828–35. 10.1093/scan/nsu020 PMC4221227 24478326

[81] Felmingham K Williams LM Kemp AH Liddell B Falconer E Peduto A Neural responses to masked fear faces: sex differences and trauma exposure in posttraumatic stress disorder. J Abnorm Psychol (2010) 119(1):241–7. 10.1037/a0017551 20141261

[82] Maheu FS Dozier M Guyer AE Mandell D Peloso E Poeth K A preliminary study of medial temporal lobe function in youths with a history of caregiver deprivation and emotional neglect. Cognit Affect Behav Neurosci (2010) 10(1):34–49. 10.3758/CABN.10.1.34 20233954 PMC2926942

[83] Garrett AS Carrion V Kletter H Karchemskiy A Weems CF Reiss A Brain activation to facial expressions in youth with PTSD symptoms. Depress Anxiety (2012) 29(5):449–59. 10.1002/da.21892 PMC6712984 22553009

[84] Phelps EA Human emotion and memory: interactions of the amygdala and hippocampal complex. Curr Opin Neurobiol (2004) 14(2):198–202. 10.1016/j.conb.2004.03.015 15082325

[85] Herman JP Ostrander MM Mueller NK Figueiredo H Limbic system mechanisms of stress regulation: hypothalamo-pituitary-adrenocortical axis. Prog Neuropsychopharmacol Biol Psychiatry (2005) 29(8):1201–13. 10.1016/j.pnpbp.2005.08.006 16271821

[86] Kiem SA Andrade KC Spoormaker VI Holsboer F Czisch M Samann PG Resting state functional MRI connectivity predicts hypothalamus-pituitary-axis status in healthy males. Psychoneuroendocrinology (2013) 38(8):1338–48. 10.1016/j.psyneuen.2012.11.021 23279846

[87] Pruessner JC Dedovic K Khalili-Mahani N Engert V Pruessner M Buss C Deactivation of the limbic system during acute psychosocial stress: evidence from positron emission tomography and functional magnetic resonance imaging studies. Biol Psychiatry (2008) 63(2):234–40. 10.1016/j.biopsych.2007.04.041 17686466

[88] Novak LR Gitelman DR Schuyler B Li W Olfactory-visual integration facilitates perception of subthreshold negative emotion. Neuropsychologia (2015) 77:288–97. 10.1016/j.neuropsychologia.2015.09.005 PMC4699288 26359718

[89] Dominguez-Borras J Rieger SW Corradi-Dell’Acqua C Neveu R Vuilleumier P Fear Spreading Across Senses: Visual Emotional Events Alter Cortical Responses to Touch, Audition, and Vision. Cereb Cortex (2017) 27(1):68–82. 10.1093/cercor/bhw337 28365774 PMC5939199

[90] Bertsch K Gamer M Schmidt B Schmidinger I Walther S Kastel T Oxytocin and reduction of social threat hypersensitivity in women with borderline personality disorder. Am J Psychiatry (2013) 170(10):1169–77. 10.1176/appi.ajp.2013.13020263 23982273

[91] Schneider I Boll S Volman I Roelofs K Spohn A Herpertz SC Oxytocin Normalizes Approach-Avoidance Behavior in Women With Borderline Personality Disorder. Front Psychiatry (2020) 11:120:120. 10.3389/fpsyt.2020.00120 32218744 PMC7078372

[92] Bakermans-Kranenburg MJ van IJMH Sniffing around oxytocin: review and meta-analyses of trials in healthy and clinical groups with implications for pharmacotherapy. Transl Psychiatry (2013) 3:e258. 10.1038/tp.2013.34 23695233 PMC3669921

[93] Riem MM van IMH Tops M Boksem MA Rombouts SA Bakermans-Kranenburg MJ Oxytocin effects on complex brain networks are moderated by experiences of maternal love withdrawal. Eur Neuropsychopharmacol (2013) 23(10):1288–95. 10.1016/j.euroneuro.2013.01.011 23453164

[94] Croy I Schellong J Joraschky P Hummel T PTSD, but not childhood maltreatment, modifies responses to unpleasant odors. Int J Psychophysiolol (2010) 75(3):326–31. 10.1016/j.ijpsycho.2010.01.003 20079770

[95] Colman I Kingsbury M Garad Y Zeng Y Naicker K Patten S Consistency in adult reporting of adverse childhood experiences. Psychol Med (2016) 46(3):543–9. 10.1017/s0033291715002032 26511669

[96] MacDonald K Thomas ML Sciolla AF Schneider B Pappas K Bleijenberg G Minimization of Childhood Maltreatment Is Common and Consequential: Results from a Large, Multinational Sample Using the Childhood Trauma Questionnaire. PloS One (2016) 11(1):e0146058. 10.1371/journal.pone.0146058 26815788 PMC4729672

