# Supplementary material for: Childhood Maltreatment Alters the Neural Processing of Chemosensory Stress Signals
Source: Front Psychiatry. 2020 Aug 6;11:783. doi: 10.3389/fpsyt.2020.00783 (PMC7425696; doi:10.3389/fpsyt.2020.00783)
Supplement: Supplementary file 1 [file DataSheet_1.docx]

**SUPPLEMENTARY INFORMATION**

Childhood maltreatment alters the neural processing of chemosensory stress signals

Ayline Maier, Luca Heinen-Ludwig, Onur Güntürkün, René Hurlemann, and Dirk Scheele

**SUPPLEMENTARY METHODS**

**Ethics and enrollment**

The study protocol was approved by the local ethics committee of the Medical Faculty of the University of Bonn, Germany. The study was registered in the Clinical Trials.gov database (Identifier: NCT03265899) provided by the US National Institutes of Health. All participants gave written informed consent and the study was conducted in accordance with the latest revision of the Helsinki Declaration. Participants were recruited from the local population by means of online advertisement and public postings. After completion of the study, participants received monetary compensation. The random allocation sequence (for the double-blind, cross-over oxytocin/placebo treatment) was generated by D.S. A.M. enrolled all participants and assigned participants to the treatment based on the random allocation plan. All behavioral and fMRI data were collected in Bonn, Germany.

**Study Design**

For the experimental session, participants were asked to maintain their regular sleep and waking times and to abstain from caffeine and alcohol intake for 24 hours prior to study arrival. This was verified via an informational questionnaire administered at the beginning of each testing session. At the beginning of each testing session, all female participants were required to undergo a urine pregnancy test prior to nasal spray administration and to confirm that they did not use hormonal contraception. Furthermore, to avoid hormonal interferences with olfactory functioning and the OXT system, all female participants were tested in their luteal phase (1).

**Axillary Sweat Collection**

Axillary sweat samples were collected from an independent sample of 30 healthy male donors (mean ± SD age, 23.30 ± 2.67 years) prior to the data collection of the present study. Sweat donors underwent both a bicycle ergometer training at a constant pulse rate of 130 BPM (sport sweat) and the Trier Social Stress Test (TSST; stress sweat) (2) in two separate 25 min-sessions. All donors were non-smokers and reported no history of somatic or mental disorders, use of medication, and drug or alcohol abuse, as assessed by the Mini-International Neuropsychiatric Interview (M.I.N.I.) (Sheehan *et al*, 1998). All donors were instructed to follow a strict protocol to prevent body odor contamination 24 h before the sweat collection sessions, which included refraining from odorous food (garlic, asparagus, heavily-flavored food), alcohol, caffeine, excessive exercise, and perfumed toiletries (perfumes, deodorants, antiperspirants, aftershaves, body lotions, shower gels or hair products). On the sweat sampling days, donors were instructed to take a shower with a scent-free shower gel (Sensiva Washing Lotion; Schülke & Mayr GmbH, Norderstedt, Germany) that was provided by the experimenter two days prior to the first testing session and to only wear loose and odorless clothes. During the donation sessions, subjects wore tight cotton t-shirts provided by the experimenter that were washed with a scent-free detergent. This sweat donation study was carried out in accordance with the latest Declaration of Helsinki principles and approved by the institutional ethics committee. Written informed consent was obtained from all donors (for full description, see (1)).

**Olfactory Stimuli**

Odor stimuli were gathered from donors’ axillae by attaching clean 6.5 x 4.3 x 4.3 inches cotton nursing pads (NUK Ultra Thin Disposable Nursing Pads; MAPA GmbH, Zeven, Germany) using surgical tape (Leukosilk^®^; BSN Medical GmbH, Luxembourg). Sweat pads were removed immediately after stressor termination, cut into eight pieces of the same size and subsequently pooled and stored at -80°C. Each olfactory sweat stimuli set (stress sweat, sport sweat) was generated 60 min prior to fMRI scanning by compiling four sweat pad pieces from four different donors of each sweat donation session.

**FMRI Task**

Facial stimuli of two male actors were obtained from the set of Karolinska Directed Emotional Faces (KDEF) database (3) and morphed from neutral (0%) to fearful expression (100%) applying 5% increments using a morphing software (FantaMorph, Abrosoft). For the emotion recognition forced-choice task, 0% (neutral), 30% (low fearful), 40% (medium fearful) and 100% (high fearful) were used. The resulting eight images were presented 18 times each during the experiment in a random order.

Visual stimuli were presented on a white backdrop on a 32-inch MRI compatible TFT LCD monitor (NordicNeuroLab, Bergen, Norway) positioned at the rear end of the magnet bore using Presentation 14 (Neurobehavioral Systems, Albany, CA). Participants completed the fMRI task on an MRI-compatible response grip system (**NordicNeuroLab AS,** Bergen, Norway).

**fMRI Data Analysis**

***Preprocessing***

The MRI data were preprocessed and analyzed using SPM12 software (Wellcome Trust Centre for Neuroimaging, London, United Kingdom; http://www.fil.ion.ucl.ac.uk/spm) implemented in MATLAB R2010b (MathWorks, Natick, Massachusetts). The first five volumes of each functional time series were discarded to allow for T1 equilibration. Images were corrected for head movement between scans by an affine registration. For realignment, a two-pass procedure was used, by which images were initially realigned to the first image of the time series and subsequently re-realigned to the mean of all images. For normalization, a two-step procedure was applied. Normalization parameters were first determined by segmenting the T1-image using the default tissue probability maps. Next, normalization parameters were applied to normalize the functional images to the standard anatomical Montreal Neurological Institute (MNI) space resampled at a 2 × 2 × 2 mm voxel. The normalized images were spatially smoothed using a 6-mm FWHM Gaussian kernel. Raw time series were detrended using a high-pass filter (cut-off period, 128 s).

***First-Level analysis***

On the first level, onsets and durations of the 24 experimental conditions (‘Sport_Neutral_OXT_’, ‘Stress_Neutral_OXT_’, ‘Raspberry_Neutral_OXT_’, ‘Sport_LowFearful_OXT_’, ‘Stress_LowFearful_OXT_’, ‘Raspberry_LowFearful_OXT_’, ‘Sport_MediumFearful_OXT_’, ‘Stress_MediumFearful_OXT_’ ‘Raspberry_MediumFearful_OXT_’, ‘Sport_HighFearful_OXT_’, ‘Stress_HighFearful_OXT_’, ‘Raspberry_HighFearful_OXT_’, ‘Sport_Neutral_PLC_’, ‘Stress_Neutral_PLC_’, ‘Raspberry_Neutral_PLC_’, ‘Sport_LowFearful_PLC_’, ‘Stress_LowFearful_PLC_’, ‘Raspberry_LowFearful_PLC_’, ‘Sport_MediumFearful_PLC_’, ‘Stress_MediumFearful_PLC_’ ‘Raspberry_MediumFearful_PLC_’, ‘Sport_HighFearful_PLC_’, ‘Stress_HighFearful_PLC_’, ‘Raspberry_HighFearful_PLC_’) were modeled using a stick function convolved with a hemodynamic response function, with the trial onset defined as the onset of odor delivery. Respiratory noise correction was performed using the PhysIO toolbox (4) by computing RETROICOR (retrospective image correction) regressors (5) using a 4th order Fourier expansion for the respiratory phase (6) and RVT (respiratory volume per time) (7) regressors. The movement parameters (realignment parameters) and respiratory noise regressors were included as nuisance regressors in the design matrix. For the fMRI statistical analysis, we used a two level random-effects approach based on the general linear model as implemented in SPM12 (see (1)).

**SUPPLEMENTARY RESULTS**

**Behavioral Results**

Regression analysis demonstrated no significant association between CTQ sum scores and stress-specific emotion recognition scores (i.e. stress – sport condition) for facial stimuli exhibiting a neutral (*β* = -0.15, *P* = 0.273), low fearful (*β* = -0.19, *P* = 0.145) and medium fearful expression (*β* = 0.01, *P* = 0.915) under PLC. Furthermore, CTQ sum scores did not predict the modulatory effect of OXT on stress-specific emotion recognition scores for facial stimuli exhibiting neutral (*β* = -0.05, *P* = 0.735), low fearful (*β* = -0.2, *P* = 0.143), medium fearful (*β* = 0.12, *P* = 0.388) or high fearful expressions (*β* = 0.17, *P* = 0.214). Moreover, CTQ sum scores did not predict emotion recognition scores for neutral (*β* = -0.15, *P* = 0.272), low fearful (*β* = -0.13, *P* = 0.314), medium (*β* = -0.24, *P* = 0.072) and high fearful facial expressions (*β* = -0.08, *P* = 0.537) when subjects were exposed to the non-social control odor raspberry in the PLC condition. However, CTQ sum scores predicted the emotion recognition scores for low fearful facial expressions (*β* = -0.34, *P* = 0.01), medium fearful facial expressions (*β* = -0.28, *P* = 0.033), but not for neutral (*β* = -0.135, *P* = 0.318) and high fearful expressions (*β* = -0.01, *P* = 0.948) when subjects were exposed to the non-social control odor raspberry in the OXT condition.

Correlation analyses showed no significant associations between CTQ sum scores and post fMRI pleasantness, intensity and familiarity ratings for sport odor stimuli after they received intranasal PLC (pleasantness: *r* = 0.38, *P* = -0.119; intensity: *r* = 0.16, *P* = 0.243; familiarity: *r* = -0.1, *P* = 0.44) and after they received intranasal OXT (pleasantness: *r* = 0.08, *P* = 0.531; intensity: *r* = 0.051, *P* = 0.706; familiarity: *r* = -0.05, *P* = 0.717). Likewise, we did not observe significant associations between CTQ sum scores and post fMRI pleasantness, intensity and familiarity ratings for stress odor stimuli in the PLC condition (pleasantness*: r* = -0.16, *P* = 0.24; intensity: *r* = 0.1, *P* = 0.478; familiarity: *r* = -0.16, *P* = 0.245) and in the OXT condition (pleasantness: *r* = 0.11, *P* = 0.433; intensity: *r* = -0.18, *P* = 0.178; familiarity: *r* = -0.05, *P* = 0.717). Moreover, we did not detect significant associations between CTQ sum scores and post fMRI pleasantness, intensity and familiarity ratings for the non-social control odor raspberry in the PLC condition (pleasantness: *r* = -13, *P* = 0.325; intensity: *r* = -0.14, *P* = 0.286; familiarity: *r* = 0.12, *P* = 0.368) and in the OXT condition (pleasantness: *r* = -0.1, *P* = 0.932; intensity: *r* = -0.09, *P* = 0.484; familiarity: *r* = 0.014, *P* = 0.915).

**fMRI Results**

Childhood maltreatment was significantly associated with amygdala and hippocampus responses to stress relative to sport odor cues, but exploratory analyses showed no significant correlation between parameter estimates of amygdala and hippocampus responses to stress and sport odor cues compared to baseline (cf. Figure S2).

**SUPPLEMTARY FIGURES**

**Figure S1**

**
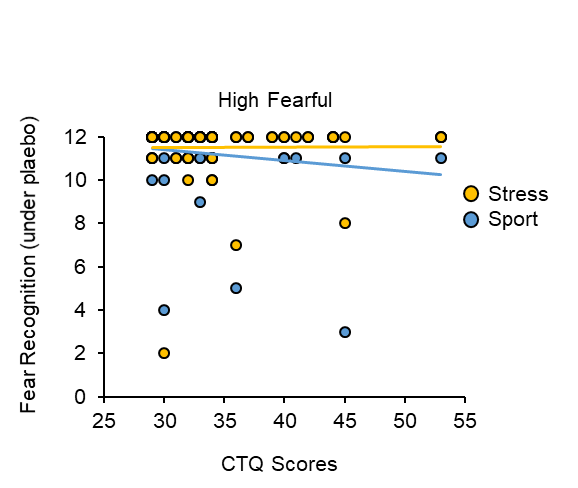
**

Childhood maltreatment and the recognition of high fearful facial stimuli during the exposure to stress odor and sport odor. CTQ, Childhood Trauma Questionnaire.

**Figure S2**

**
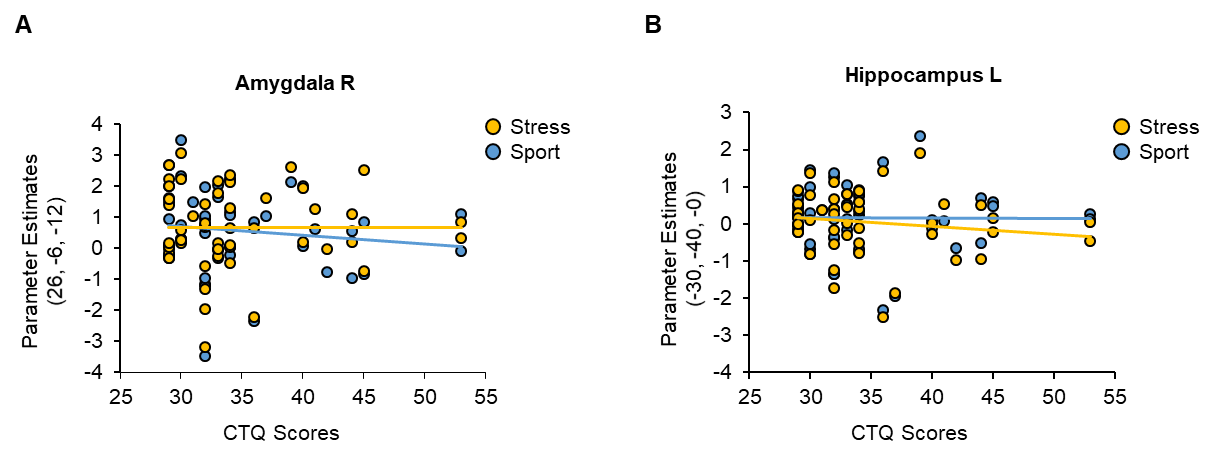
**

Childhood maltreatment and impact of stress odor and sport odor on **(A)** amygdala and **(B)** hippocampal activity. CTQ, Childhood Trauma Questionnaire.


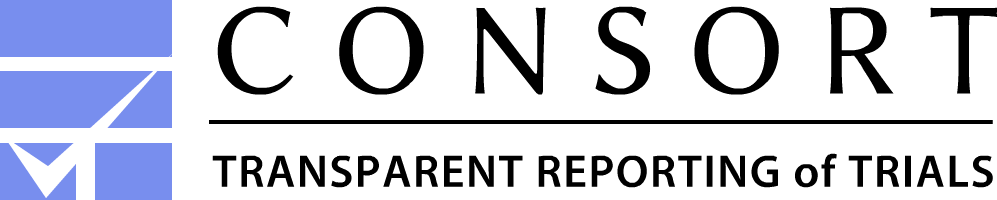


**CONSORT 2010 Flow Diagram**

Analysed behavioral data (n= 58)

Analysed MRI data (n= 50)

♦ Excluded from MRI analysis (technical malfunctions or excessive head motion > 3 mm/º during scanning) (n= 8)

Analysed behavioral data (n= 58)

Analysed MRI data (n= 50)

♦ Excluded from MRI analysis (technical malfunctions or excessive head motion > 3 mm/º during scanning) (n= 8)

**Follow-Up**

**Enrollment**

Randomized (n= 58)

Assessed for eligibility (n= 60)

**Analysis**

**Allocation**

Allocated to intervention (placebo; n= 58)

♦ Received allocated intervention (n= 58)

♦ Did not receive allocated intervention (give reasons) (n= 0)

Lost to follow-up (give reasons) (n= 0)

Discontinued intervention (n= 0)

Allocated to intervention (oxytocin; n= 58)

♦ Received allocated intervention (n= 58)

♦ Did not receive allocated intervention (give reasons) (n= 0)

Lost to follow-up (give reasons) (n= 0)

Discontinued intervention (n= 0)

Excluded (n= 2)

♦  Did not meet inclusion criteria (n= 2)

♦  Declined to participate (n= 0)

♦  Other reasons (n= 0)

**Supplemental References**

1. Maier A, Scheele D, Spengler FB, Menba T, Mohr F, Gunturkun O, et al. Oxytocin reduces a chemosensory-induced stress bias in social perception. *Neuropsychopharmacology* (2019) 44(2):281-8. doi: 10.1038/s41386-018-0063-3

2. Kirschbaum C, Pirke KM, Hellhammer DH. The Trier Social Stress Test - a Tool for Investigating Psychobiological Stress Responses in a Laboratory Setting. *Neuropsychobiology* (1993) 28(1-2):76-81. doi: 10.1159/000119004

3. Lundqvist D, Flykt A, Öhman A. *The Karolinska Directed Emotional Faces - KDEF, CD ROM from Department of Clinical Neuroscience* Psychology section Karolinska Institutet: Psychology section Karolinska Institutet (1988).

4. Hayes AF. *Introduction to mediation, moderation, and conditional process analysis: A regression based approach*. New York, NY: Guilford Press (2013).

5. Glover GH, Li TQ, Ress D. Image-based method for retrospective correction of physiological motion effects in fMRI: RETROICOR. *Magn Reson Imaging* (2000) 44(1):162-7. doi 10.1002/1522-2594(200007)44:1<162::aid-mrm23>3.0.co;2-e

6. Harvey AK, Pattinson KT, Brooks JC, Mayhew SD, Jenkinson M, Wise RG. Brainstem functional magnetic resonance imaging: disentangling signal from physiological noise. *J Magn Reson Imaging* (2008) 28(6):1337-44. doi: 10.1002/jmri.21623

7. Birn RM, Smith MA, Jones TB, Bandettini PA. The respiration response function: the temporal dynamics of fMRI signal fluctuations related to changes in respiration. *Neuroimage* (2008) 40(2):644-54. doi: 10.1016/j.neuroimage.2007.11.059
